# Degenerative Temporomandibular Disorders: An Assessment of Bone Trabecular Structure Using Fractal Analysis in Digital Panoramic Radiographs

**DOI:** 10.7759/cureus.57449

**Published:** 2024-04-02

**Authors:** Mouttoukichenin Surenthar, Subramanian V Srinivasan, Djeapragassam Parimala, Vineeth Ramanathan

**Affiliations:** 1 Oral Medicine and Radiology, Saveetha Dental College and Hospital, Chennai, IND; 2 Oral Medicine and Radiology, Mahatma Gandhi Postgraduate Institute of Dental Sciences, Puducherry, IND; 3 Oral Medicine and Radiology, Private Practitioner, Dindigul, IND

**Keywords:** digital panoramic radiograph, bone trabeculae, fractal analysis, osteoarthritis, temporomandibular disorder

## Abstract

Introduction

Fractal analysis has proved to be a salient tool to quantitatively assess the qualitative changes in the bone trabeculae of patients with hyperparathyroidism, osteoporosis, and various temporomandibular disorders, including osteoarthritis (OA) and rheumatoid arthritis of temporomandibular joint (TMJ), in several previous studies. The purpose of this study was to use fractal analysis to assess alterations in the trabecular pattern of the mandibular condyle in patients with degenerative temporomandibular disorders.

Materials and methods

This study comprised 98 subjects with 49 subjects in the study group and 49 subjects in the control group, aged 18-50 years. Age and sex in the control group were matched to those in the case group. The subjects were assessed clinically with the Diagnostic Criteria for Temporomandibular Disorders. Digital panoramic X-ray equipment with set parameters of 70 kvp, 8 mA, and 16-second exposure duration was used to take panoramic radiographs. Fractal analysis was done and the calculated fractal dimension value was obtained using ImageJ version 1.48 software (National Institutes of Health, Bethesda, MD). The same observer used Muir and Goss's method to rate the total degenerative changes in the condylar surfaces, which were substantiated by the calculated fractal dimension value. The data were statistically analyzed.

Results

The results revealed a significant difference (p-value = 0.041) between the mean fractal value in the case group's (1.35) and the control group's (1.38) left sides whereas the differences in the mean fractal values between the case and control groups on the right side was not significant (p-value = 0.49).

Conclusion

It is recommended to use the fractal dimension value and the total degenerative severity score together to quantify degenerative changes in the TMJ OA rather than exclusively relying on fractal value.

## Introduction

Osteoarthritis (OA) or osteoarthrosis, which are other names for degenerative joint disease, is a degenerative disorder of the joint that is characterized by the degeneration of articular tissue along with concurrent osseous alterations in the condyle and/or articular eminence. Almost 10% to 17% of patients with temporomandibular joint (TMJ) pain have OA and the overall prevalence of OA in both clinical and MRI examinations between 20 and 49 years of age is 25%. Clinically, it manifests with joint pain, restricted mouth opening, and fine and coarse crepitations palpated during centric and eccentric jaw movements [1].

Indeed, imaging in TMJ OA is crucial since it is regarded as the diagnostic reference standard. Osseous changes in TMJ OA due to progressive, regressive, and circumferential articular remodeling include erosion, flattening, sclerosis, osteophyte formation, subcortical cyst, resorption of the head of the condyle, and reduced articular space [2]. Although CT is the most efficient in detecting osseous changes in TMJ OA as described by the Diagnostic Criteria for Temporomandibular Disorders (DC/TMD; 2012) [3], panoramic radiographs being the most routinely and widely used diagnostic tool by clinicians can very well aid in appreciating the degenerative changes within the joint components in OA [4].

Fractal analysis (FA) is a mathematical technique for evaluating irregular, self-similar, and complex body structures as they pose difficulty in assessment and evaluation by conventional methods. The quantitative outcome obtained through FA is known as the fractal dimension (FD) [5]. FA is carried out in digitalized images using ImageJ software (National Institutes of Health, Bethesda, MD) and the box-counting method. Temporomandibular disorders (TMDs) were evaluated with FA in panoramic radiographs and have shown reliable results. Further, similar studies have been done in OA of the knee, wherein the FA of trabecular bone is considered to be a more sensitive marker than bone mineral density for evaluating the disease status [6].

Although the prognosis of TMJ OA is relatively good owing to the functional remodeling of the joint surfaces and adaptive capacity of the articular disc, severe forms of unilateral TMJ OA can result in idiopathic condylar resorption leading to loss of posterior support in the affected condyle with an ipsilateral deviation of the mandible thus collapsing the entire masticatory balance [7], highlighting the purpose and significance of this study.

Since bone trabeculae is a complex geometric structure and this complexity is disfigured in degenerative temporomandibular diseases, the purpose of our study was to assess the level of aforesaid bone trabecular changes in the subchondral bone (i.e., head of the condyle) using FA. This study is unique in the way that early degenerative changes, which were believed to be unreflective in a panoramic radiograph, can still be utilized to evaluate the same using a contemporary mathematical method that would serve as an alternative to other expensive, time-consuming advanced imaging modalities and also a screening tool to evaluate the severity of disease using calculated fractal values.

## Materials and methods

The present study was conducted following approval from the Mahatma Gandhi Postgraduate Institute of Dental Sciences, Pondicherry (4032020) at the XXI Research and Ethical Committee Meeting. This study comprised 98 subjects with 49 subjects in the study group and 49 subjects in the control group, in the age range of 18-50 years, randomly selected from the outpatient department of oral medicine and radiology by simple random sampling method. It was a single-centered, analytical, cross-sectional imaging study. Following thorough clinical examination, the subjects were included in the study group after informed consent and screening for degenerative changes in the TMJ in the digital panoramic radiographs. The study group consisted of patients between the ages of 18 and 50 years who were diagnosed with degenerative TMD but otherwise were in good systemic health. The control group was made up of normally healthy patients whose age and sex were matched to that of the study group. Panoramic radiographs were requested for a variety of dental issues, including dental caries, periodontitis, and impacted teeth. The maximum of lost premolar/molar teeth in each quadrant among the subjects in either group was one. Individuals with conditions and those under medications, affecting bone metabolism, fractures involving condyle, and those who had already received conservative or surgical treatment for TMD in either group were excluded.

After a thorough clinical examination recommended by the DC/TMD, digital panoramic radiographs were advised to screen for degenerative changes in the TMJ. Digital panoramic X-ray equipment with set parameters of 70 kvp, 8 mA, and 16-second exposure duration was used to take panoramic radiographs. After the exposure, the radiographic images were displayed on a 24.1-inch wide, flat-panel liquid crystal display (LCD) color screen with 1366 x 768 resolution under dim, quiet lighting conditions.

The images were initially checked out and those free of positional errors, superimpositions, and artifacts were evaluated for degenerative changes such as flattening, sclerosis, erosion, osteophyte formation, subcortical cyst, resorption of the head of the condyle, and reduced articular space. Those radiographs with the degenerative changes were included in the study group as diagnosed cases of degenerative joint disease of TMJ. Based on the inclusion criteria outlined above, control group radiographs from patients who were systemically healthy and requiring panoramic radiographs for various dental pathologies like multiple caries, impacted third molars, etc. were obtained by matching the age and gender with those of the study group. The digital radiographs were exported in the bitmap image file format from the Planmeca Romexis® dental imaging software (Planmeca, Helsinki, Finland). The radiographic images from both the study and control groups were then analyzed fractally.

**Figure 1:**
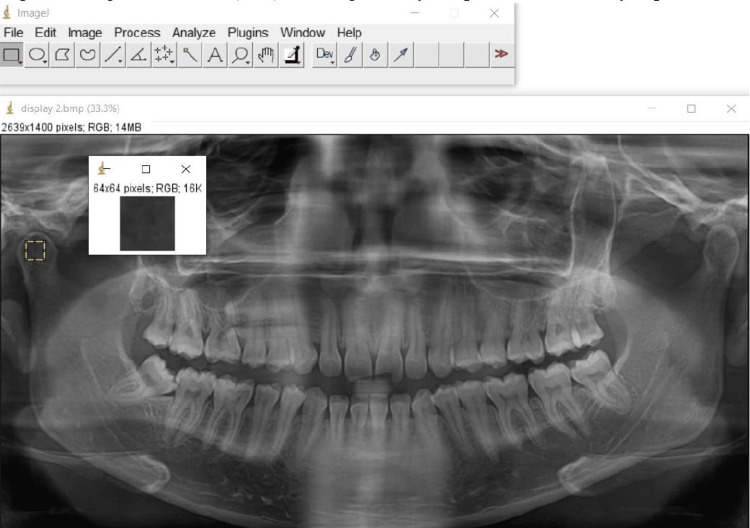
Region of interest (ROI) selected (yellow squares) within the right condyle is processed further by duplication.

**Figure 2:**
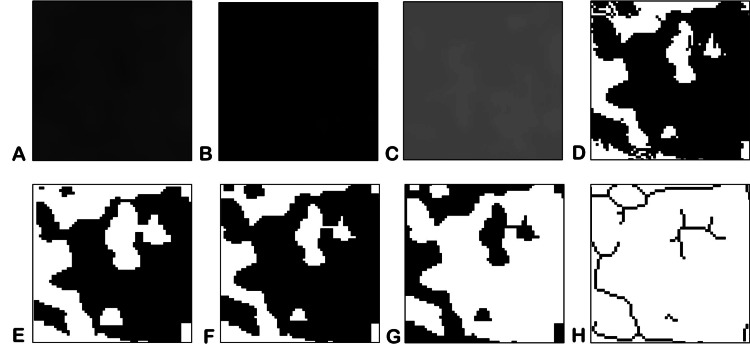
Sequence of functions used for fractal analysis. A: Gaussian blur. B: The region of interest is subtracted from the background. C: A 128-gray value is added to each pixel location. D: Binarization. E: Erosion. F: Dilatation. G: Inversion. H: Skeletonization.

**Figure 3:**
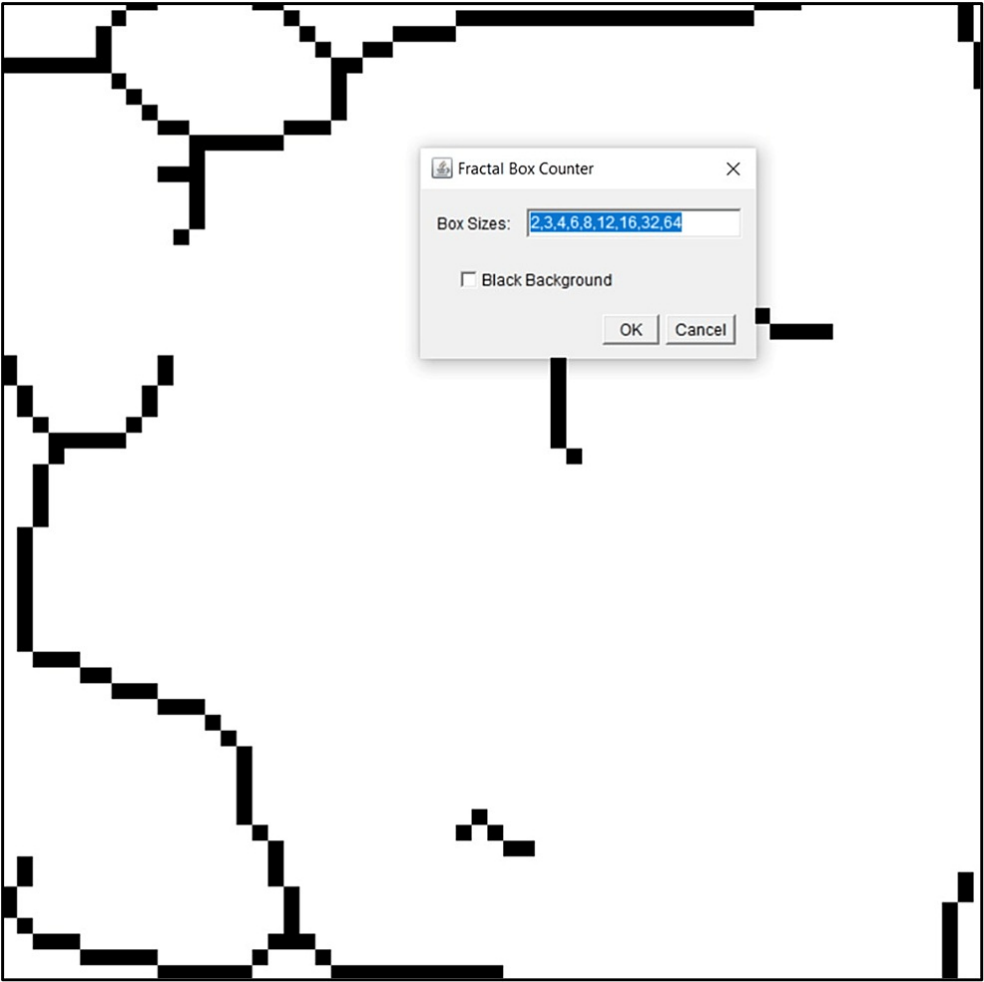
The box-counting algorithm is applied to the skeletonized image.

**Figure 4:**
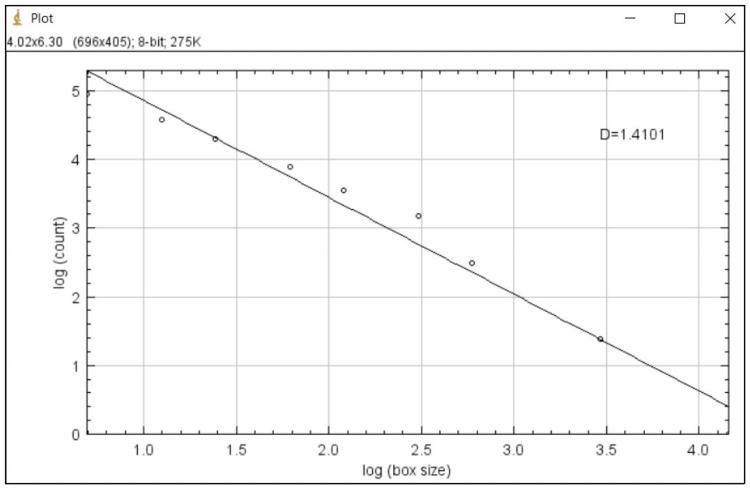
Calculation of fractal dimension (FD).

The fractal analysis was performed by the observer with skills in dentomaxillofacial radiology using the free and open-source public domain software ImageJ (64-bit Java version 1.8.0_172 for Windows, United States), which was downloaded from https://imagej.nih.gov/ij/download.html. At first, standardized regions of interest (ROI) in the shape of squares of 64 x 64 pixel size were selected in the subcondylar cancellous bone just interior to the cortex of the right and left mandibular condyles close to the articulating surface based on the average surface area of the cancellous bone accessible beneath the cortex of the condylar head and this size was adhered to for all radiographs. Cancellous bone is characterized by a very porous texture and a complex network of interconnecting trabeculae. It is preferred over corticated bone because of its complexity, which is well-suited for fractal analysis. The method proposed by White and Rudolph was used for fractal analysis and the steps involved in the calculation of FD were extracted from the study by Arsan et al. [8], as represented in Figures 1-4.

After selecting the ROI, the images were duplicated for further processing (Figure 1). Soft tissues overlying the images were blurred with Gaussian blur to eliminate variations in brightness (Figure 2A). After that, the resultant image was subtracted from the original (Figure 2B). With the addition of 128 grey values to each pixel location, bone marrow spaces and trabeculae were distinguished from one another (Figure 2C). The binarization function was used to outline bone marrow spaces and trabeculae (Figure 2D). The resulting image was eroded to remove noise (Figure 2E) and the dilation function was used to emphasize the structural outlines (Figure 2F). The trabeculae were made black and the bone marrow spaces were made white with the help of inversion (Figure 2G). The image was then skeletonized to prepare the ROI for fractal analysis (Figure 2H). The box-counting method was employed by applying 2-64 pixels dimensions over the ROI using the box-counting algorithm available under the toolbar of the software (Figure 3). A guide with boxes varying in size from 2 to 64 pixels was superimposed on the ROI in the box-counting technique. A count was done for every series of different-sized boxes included in the binary image outline. Trabeculae were counted and plotted against box sizes on a graphical scale of logarithmic proportions. The slope of the line was used to compute the fractal dimension (D) of the ROI selected, which gives the D value for the condylar trabecular structures within that ROI (Figure 4).

According to the box-counting method followed, the FD is the number of boxes needed to cover the trabecular pattern. A higher FD reveals a more complex trabecular pattern. Muir and Goss's method [9] was used by the same observer to rate the type and severity of degenerative changes in the condylar surfaces. A scoring was given for each degenerative change from 0 to 2, with 0 demonstrating no change, 1 showing mild change, and 2 when there is a gross change. The overall scoring of all the changes for each condyle was summed up and compared between the case and control groups to check for significant differences to substantiate changes in FD value.

Statistical analysis

IBM SPSS version 20.0 (IBM Corp., Armonk, NY) was used to analyze descriptive and inferential statistics. To summarize quantitative data comparing main groups and subgroups, mean and standard deviation were employed. The Mann-Whitney U test for independent samples was employed to compare quantitative data between groups. Throughout the investigation, a difference was deemed statistically significant if the p-value was less than 0.05.

## Results

Table 1 shows the distribution of age and gender among participants in the study. There were 22 female participants (44.9%) and 27 male participants (55.1%) in the case group. In the control group, there were 26 male participants (53.1%) and 23 female participants (46.9%). The mean age was 26.5 in the case group and 27.6 in the control group, respectively.

**Table 1:** Age and gender distribution of the study participants (N = 98).

Variable	Gender	Cases		Control	
		N	%	N	%
Gender	Female	22	44.9	23	46.9
	Male	27	55.1	26	53.1
Age		Mean	SD	Mean	SD
		26.4694	8.62434	27.5918	6.54446

Table 2 describes the comparison of the case and control groups concerning the right side. The Mann-Whitney U test revealed no significant difference (p-value = 0.49) between the fractal values on the right sides of the case group and the control group, but when degenerative changes like osteophyte (p-value = 0.013) and sclerosis (p-value = 0.002) were compared, there was a significant difference between the cases and controls. The total degenerative change score between the case and control groups had a significant difference with a p-value of 0.001 on the right side.

**Table 2:** Comparison of the right side of the case group and the right side of the control group.

Variables	Group	Mean	Std. deviation	Mean rank	Sum of rank	p-value
Fractal value	Control	1.35	0.08	42.09	2062.50	0.49
	Case	1.31	0.08	31.98	863.50	
Osteophyte	Control	0.02	0.14	37.78	1851.00	0.25
	Case	0.07	0.27	39.81	1075.00	
Erosion	Control	0.16	0.47	35.17	1723.50	0.013
	Case	0.48	0.70	44.54	1202.50	
Flattening	Control	0.31	0.58	36.87	1806.50	0.27
	Case	0.44	0.64	41.46	1119.50	
Sclerosis	Control	0.24	0.56	33.86	1659.00	0.002
	Case	0.74	0.81	46.93	1267.00	
Concavity	Control	0.02	0.14	37.78	1851.00	0.25
	Case	0.07	0.27	39.81	1075.00	
Subcortical cyst	Control	0.14	0.50	36.58	1792.50	0.08
	Case	0.41	0.80	41.98	1133.50	
Total score	Control	0.90	1.05	30.73	1506.00	0.001
	Case	2.22	1.25	52.59	1420.00	

Table 3 describes the comparison of the case and control groups concerning the left side. Mann-Whitney U test revealed a significant difference (p-value = 0.041) between the fractal values in the case and control group's left sides. The degenerative changes such as erosion (p-value = 0.014) and sclerosis (p-value = 0.001) also showed a significant difference between the cases and controls on the left sides. There was a significant difference in the total degenerative change score between the case and control groups.

**Table 3:** Comparison of the left side of the case group and the left side of the control group.

Variables	Group	Mean	Std. deviation	Mean rank	Sum of rank	p-value
Fractal value	Control	1.38	0.08	51.32	1968.00	0.041
	Case	1.35	0.07	49.84	1844.00	
Osteophyte	Control	0.00	0.00	43.00	2107.00	0.25
	Case	0.03	0.16	44.16	1634.00	
Erosion	Control	0.06	0.32	40.32	1975.50	.014
	Case	0.24	0.49	47.72	1765.50	
Flattening	Control	0.16	0.43	42.43	2079.00	0.47
	Case	0.30	0.66	44.92	1662.00	
Sclerosis	Control	0.16	0.47	35.33	1731.00	0.001
	Case	0.73	0.73	54.32	2010.00	
Concavity	Control	0.04	0.20	42.19	2067.50	.20
	Case	0.19	0.57	45.23	1673.50	
Subcortical cyst	Control	0.14	0.50	42.03	2059.50	.25
	Case	0.27	0.65	45.45	1681.50	
Total score	Control	0.55	0.82	33.51	1642.00	0.001
	Case	1.76	1.40	56.73	2099.00	

## Discussion

In the 49 TMJ OA cases studied, there were 34 cases unilaterally affected and 15 cases bilaterally affected. In total, there were 37 affected left sides and 27 affected right sides in the case group. There was a statistically significant difference between the case group and the control group on the left side for their fractal dimension values and the severity of degenerative changes. The fractal value of the case group on the left side was significantly lower than the control group (1.35 < 1.38). Although there was no statistically significant difference between the case and control groups on the right side, the fractal value of the affected right TMJ was lower compared to the control group (1.31 < 1.35) and the severity of degenerative changes was significantly higher in the case group than in the control group. This suggests that bone microarchitecture differs considerably between TMJ OA patients and healthy individuals.

With advanced osteoarthritis, bone stiffness decreases at a fixed apparent bone volume fraction, which can be explained by a decrease in the calcium-to-collagen ratio and hypomineralization of trabecular material [10]. Thus, the micromechanical and microarchitectural properties of the subchondral bone tissue are affected in OA patients.

The concept of calculating the fractal dimension of trabecular bone patterns has been in practice both in long bones and jawbones on different digitalized radiographic images like intraoral periapical radiographs and panoramic radiographs since trabecular bone display fractal properties within a defined box size, which corresponds to the size of a structural unit of trabecular bone [11]. In their work on changes in the trabecular pattern of the jaws in individuals with osteoporosis, White and Rudolph first created a customized computed program using ImageJ software that assessed the architecture of trabecular bone in digitized conventional radiographs [12]. Later, this method was popularized on direct digital radiographs with improved preciseness. In many studies, box-counting has been used to calculate fractal dimension, and the same was applied to evaluate the trabecular pattern of mandibular condyles in the present study.

While there is a strong female preponderance for TMJ OA in the previously reported studies [13,14] attributed to polymorphism of estrogen receptor and its association with increased pain susceptibility in females [15], the present study interestingly demonstrated a slight male predilection (27 males (55.1%) and 22 females (44.9%)) in the ratio of 1.2:1. However, this is inconclusive for the sample size studied and there was no statistically significant difference too. The age range in our case group varied from 18 to 50 years with a mean age of 26.4 years. The control group was age and sex-matched with 26 males (53.1%) and 23 females (46.9%). The mean age in the control group was 27.5 years.

In the comparison between the right side of the case group and the control group (Table 2), the fractal value between the cases and controls was not statistically significant though the mean fractal value was less in the case group. There was a statistically significant difference between the case group and the control group on the left side (Table 3). Both of these findings were consistent with the study conducted by Arsan et al. [8], wherein although the fractal value of the right side in the case group was less compared to the control group, it was not statistically significant and there was a statistically significant difference on the left side supporting the present study. It was also in line with the study conducted by Yesiltepe et al. [16] on patients with rheumatoid arthritis of TMJ in which there was a statistically significant difference between the case and the control group on the right side. Also, the fractal findings were supported by the studies conducted by Gumussoy et al. [17] and Jayachandran et al. [18] done on digital panoramic radiographs in patients with chronic renal failure and rheumatoid arthritis. In all the aforementioned studies, the fractal value was significantly lower compared to their healthy controls demonstrating that fractal value decreases with demineralization and reduced bone mineral density since the complexity of the trabecular bone pattern becomes altered. The statistically insignificant difference between the case and control groups on the right side in the present study may presumably be due to the smaller number of right sides studied.

There was a statistically significant difference between the case and control groups on both sides (Tables 2, 3) in the overall degenerative score with a p-value of 0.001 exhibiting marked degenerative changes in the case group primarily contributed by the statistically significant changes in erosion and sclerosis on both sides. Flattening had no significant difference on both the sides between the case and control groups, probably it being the normal adaptive change to overloading on the articulating surfaces and it was the most common degenerative finding in the present study when both sides of the case and control groups were considered together.

There are numerous previous studies on osteoporosis and fractal analysis substantiating the fact that the FD value decreases with lower bone mineral density and bone mass in osteoporosis [12,19]. However, whether the FD value increases once these osteoporotic defects remodel and deposit bone as an adaptive mechanism is debatable. Li et al. reported that in subchondral sclerosis, subchondral bone remains hypo-mineralized despite the increase in bone volume fraction due to improper bone remodeling [20]. Kraus et al. proposed that fractal value decreased in the sclerosed and remodeled knee joint regions since the quality of the bone trabeculae remained altered with decreased complexity even though bone mineral density increased [21]. Perhaps, this had steered the overall FD value on the left side to be lower in the case group compared to the control group with a statistically significant difference. Thus, fractal analysis may be a more sensitive tool in quantifying cancellous trabecular bone than estimating bone mineral density, although the correlation of FD value with that of the degenerative changes was not achieved in the present study, which could be one of the limitations.

Limitations

The results may not be as generalizable as it is non-representative of the overall degenerative temporomandibular disorder population. Because of its cross-sectional methodology, the study may not be able to prove a link between degenerative TMDs and bone trabecular structure. The study's particular fractal analysis methodology may include assumptions or restrictions that affect the outcome. It is crucial to ensure the fractal analysis technique's validity and robustness.

Future scope

This study highlights the future scope to conduct longitudinal studies investigating alterations in the trabecular structure of the bone over time in individuals with degenerative TMDs that may yield important information about the course of the disease and the effectiveness of treatment. By combining fractal analysis of panoramic radiographs with other imaging modalities like MRI or cone-beam computed tomography, a more thorough knowledge of the alterations in the bone that are linked to degenerative TMDs may be possible. Further, exploring the application of machine learning techniques for pattern identification and automated fractal parameter analysis in panoramic radiographs could expedite the analysis procedure and enhance diagnostic precision. This method may also be recommended as a tool to appreciate the treatment responses from disease-modifying osteoarthritis drugs (DMOD) in future clinical trials on TMJ OA.

## Conclusions

Even though the present study displayed a significant difference between the case and control groups in the FD value, it is not practical to exclusively rely on this value to quantify and validate the bone trabecular microarchitecture changes in the subchondral bone on the mandibular condyles. Therefore, it is preferable to use the FD value and the total degenerative severity score together to quantify degenerative changes in the TMJ OA.
